# Health literacy and associated factors in China: findings from the Wa ethnic group

**DOI:** 10.3389/fpubh.2024.1407593

**Published:** 2024-06-24

**Authors:** Wanqiu Yang, Yi Liu, Guangjie Zhang, Yu Yao, Yanqing Wang, Dan Leng, Chaoxiao Li, Kunjie Liu, Jiazhou Liu, Yongjie Pu, Mufei Li, Borui Yang, Shuting Zhang, Di Mu, Xiangyang Zhang

**Affiliations:** ^1^School of Ethnology and Sociology, Yunnan University, Kunming, China; ^2^School of Medicine, Yunnan University, Kunming, China; ^3^Yunnan Provincial Center for Population and Health Publicity and Education, Kunming, China; ^4^School of Vocational and Continuing Education, Yunnan University, Kunming, China; ^5^CAS Key Laboratory of Mental Health, Institute of Psychology, Chinese Academy of Sciences, Beijing, China; ^6^Department of Psychology, University of Chinese Academy of Sciences, Beijing, China

**Keywords:** health literacy, associated factors, “directly advancing” ethnic groups, rural community, the Wa ethnic group

## Abstract

**Background:**

The health literacy of ethnic groups in remote areas of China is far from satisfactory. However, the health literacy of ethnic groups in China remains unclear. This study aimed to explore the health literacy of the “advancing directly” ethnic group and its influencing factors.

**Methods:**

A cross-sectional study was conducted using a staged sampling method among the Wa ethnic group, who have rapidly transitioned directly from the traditional lifestyle of slash-and-burn cultivation to modern societies. We used the Health Literacy Questionnaire (HLQ) to assess health literacy. We defined low health literacy as less than 60% of the total score and adequate health literacy as more than 80% of the total score.

**Results:**

A total of 668 individuals met the inclusion criteria and the mean age was 42.19 (SD 10.56) years. The mean HLQ total score was 29.9 (SD 10.56). The prevalence of adequate health literacy was 0.89%. There were significant differences between the low and the non-low health literacy groups in terms of gender, age, education, marital status, occupation, residing place, current smoking status, and waist circumference (all *p* < 0.05). Multiple linear regression analysis showed that women (*t* = 9·418, *p* < 0.001), older age (*B* = −0.0091, *t* = −2.644, *p* = 0.008), low educational level (*B* = 0.766, *t* = 6.018, *p* < 0.001), current smoking (*B* = −2.66, *t* = −3.038, *p* = 0.008), and residence far from township (*B* = −5.761, *t* = −4.1, *p* < 0.001) were associated with low HLQ total score.

**Conclusion:**

Our findings suggest that the health literacy of the Wa ethnic group is far from favorable. It indicates the need for increased efforts in improving the health literacy of “advancing directly” ethnic groups.

## 1 Introduction

The World Health Organization (WHO) defines health literacy (HL) as personal knowledge and competence that enables individuals to acquire, understand, evaluate, and utilize information and services to promote their health and the health of those around them (1). There is increasing evidence that HL is a vital factor in health outcomes. Limited HL was related to increased hospitalization (2, 3), lower rates of medication adherence (2, 4), decreased acceptance of preventive interventions, increased health care costs (2, 5), poorer health status (6), and a higher rate of mortality among older adults (4). At the same time, lower HL can lead to communication barriers between healthcare providers and patients, impediments to citizens’ access to health information, and inefficient self-health management (6, 7). Moreover, HL followed a social gradient that reinforced existing inequalities, such as education and health resources, as well as economic development (8). HL is a better predictor of health status than age, education, income, occupation, and cultural context (8). Therefore, HL has been identified as a policy priority in the health strategy “Together for Health 2007–2013” in European Union (9), “Health 2023” in China (10), and the WHO 2030 Agenda for Sustainable Development (11).

In China, the Chinese Ministry of Health (MOH) issued the bulletin “Chinese Resident Health Literacy—Basic Knowledge and Skills (Trial)” in 2008 (12), the first government document to specify the HL of its citizens, and also developed a Health Literacy Questionnaire (HLQ) based on the HL evaluation indicators proposed by the WHO (12). Subsequently, starting in 2008, the standardized China Health Literacy Survey (CHLS) has been conducted every 2–3 years (13). The HL level of Chinese residents has steadily increased from 6.48% in 2008 to 25.4% in 2021, with 30.70% for urban residents and 22.02% for rural residents (14). However, these are huge urban–rural disparities and ethnic disparities in HL. Some studies from remote areas have reported that the HL level of rural residents was 7.94%—16.74 (15, 16). Adequate HL levels among ethnic groups range from 1.46 to 16.5% (17, 18). Moreover, reports on HL from ethnic groups in China remain inadequate (19).

Studies in Western countries have found growing evidence of lower levels of HL among ethnic groups (20, 21). Improving HL is beneficial for helping improve health inequalities among ethnic groups, and there are currently few studies in this area among Chinese ethnic groups (19), or in Western countries (20). In remote areas of China, the HL of ethnic groups is severely lacking (19). Nine of China’s 55 ethnic groups are known as “directly advancing” ethnic groups, such as the Wa, Dulong, Lisu, Jipo, and so on. Until 1960s, these ethnic groups maintained their traditional slash-and-burn farming lifestyles and only utilized their native language daily. They lived in family units, with social structures based on the family system, and their traditional lifestyle for centuries was relatively closed off. The Chinese government has been assisting in transforming their underdeveloped economic situation, particularly since 1994, through the implementation of poverty alleviation projects aimed at helping impoverished ethnic groups break the cycle of poverty (22), and these areas have seen tremendous improvements in the economy, education, and health. However, there is no HL data on “directly advancing” ethnic groups. To help fill the evidence gap, we surveyed the Wa ethnic group, one of the “directly advancing” ethnic groups in the southwest of China. The purpose of this study aimed to (1) estimate the HL level among the Wa ethnic group, and (2) assess risk factors for HL among the Wa ethnic group.

## 2 Materials and methods

### 2.1 Research design

This survey was conducted in Canyuan County, Yunnan Province, from December 2022 to February 2023. Canyuan County, the largest Wa ethnic group settlement in China, is situated on the border with Myanmar. The Wa ethnic group had preserved a slash-and-burn farming traditional lifestyle, heavily relying on family-based manual farming until 1960s.

### 2.2 Sample and setting

This was a cross-sectional study using a multi-stage cluster sampling method adopted in selecting the respondents. According to the principles of CHLS (13), the sample of the county was usually selected from six to eight administrative villages, and then two natural villages were selected from each administrative village. In this study, there were four stages for collecting the sampling. First, we randomly selected eight administrative villages from the 90 villages in Canyuan County. Second, two natural villages were selected from each administrative village, for a total of 16 natural villages. Third, using the systematic sampling method, 25 to 50 households randomly each natural village according to the size of the village. Finally, according to the KISH table (Kish Grid sampling), one household member was selected from each household. The sample size of respondents was calculated by the following formula (23). N = Z^2^P(1−P)/E^2^. Z is the significance level value (α = 0.05); P is the percentage of basic HL; E represents the maximum permissible error. The sample rate for monitoring the HL level of ethnic group residents in Yunnan province in 2019 was 12.21% (17); on this basis, a 95% confidence limit was set, with a relative error rate of no more than 20%, and E = 2%. Similarly, the minimum sample size was calculated as 525 using this formula. Meanwhile, the sample size was estimated as 577 based on a rejection rate of no more than 10%. In addition, the ideal sample size for a preliminary survey should be 5–10 times larger than the sample size in the questionnaire (24). The questionnaire consisted of 50 items; therefore, the sample size was consistent with the ideal sample size.

The inclusion criteria for this study were: (1) aged between 15 and 69; (2) local Wa ethnic residents; (3) lived in Canyuan County in the past 12 months. Exclusion criteria were: (1) providing unreliable results (*n* = 7); (2) unfinished interviews or difficulty in interviewing (*n* = 10); and (3) refusal to participate in this study (*n* = 3). The final sample consisted of 668 participants, including 353 males and 315 females. The questionnaire’s validity rate was 95%. Face-to-face interviews were conducted by trained interviewers because most respondents had low levels of education or were illiterate.

### 2.3 Ethical considerations

The study was approved by the Review Board (IRB) of the Institute of Psychology, Chinese Academy of Sciences (No. H18031), and was performed in accordance with the Declaration of Helsinki. Written informed consent was obtained prior to data collection.

### 2.4 Data collection and measurement

Demographic and socioeconomic variables were collected for all participants, including age, gender, marital status, ethnicity, education level, annual income, occupation, chronic disease, body mass index (BMI), and waist circumference (WC; in centimeters). The study used the WHO classification of general obesity and central obesity. General obesity is determined by BMI (kg/m^2^). WHO classifies BMI as: underweight = BMI < 18.5 kg/m^2^; normal = BMI 18.5–24.99 kg/m^2^; overweight = BMI 25–26.99 kg/m^2^; and obese = BMI ≥ 27 kg/m^2^ (25). In the Chinese classification, BMI is classified as underweight (BMI < 18), normal (18 ≤ BMI < 24), overweight (24 ≤ BMI < 28), or obese (BMI ≥ 28) (26). Central obesity was assessed by measuring waist circumference (WC). WC was measured at mid-breath at the level of the umbilicus with the participant in an upright position. In the WHO classification, the cut-off for central obesity is a WC of 88 cm (27). In this study, the threshold for central obesity was 90 cm WC for men and 85 cm WC for women (26).

Osborne et al. developed the Health Literacy Questionnaire (HLQ) (28), which has been translated into multiple languages for use with participants from non-English-speaking countries (29). In this study, data were obtained through face-to-face interviews, and a Chinese version of the HLQ (2022 edition) was developed by the MOH (12). It contains two parts. The first part includes mainly personal information on demographic characteristics and socioeconomic status. The second part includes 50 questions divided into 9 domains (3 aspects and 6 dimensions). The 3 aspects are health knowledge (KAA, 22 questions), health-related behaviors and lifestyles (BAL, 16 questions), and health-related skills (HRS, 12 questions). The 6 dimensions include basic and scientific health concepts (SVH, 8 questions), prevention and control of infectious diseases (ID, 6 questions), prevention and control of chronic disease (CD, 9 questions), safety and first aid (SAFA, 10 questions), medical care (MC, 11 questions), and health information (HI, 6 questions). The HL total score was calculated based on the sum of all 3 aspects or 6 dimensions. The HL total score ranged from 0 to 66. The full scores for KAA, BAL, and HRS were 28, 22, and 16, respectively, while the full scores for the 6 dimensions of SVH, ID, CD, SAFA, MC, and HI were 11, 7, 12, 14, 14, and 8, respectively. The classification was based on the 2022 Chinese criteria, a total score greater than or equal to 80% of the full score (total score ≥ 53) represents adequate HL. The cut-off points were 22, 18, and 13 for KAA, BAL, and HRS, respectively, and 9, 6,10, 11, 11, and 6 for the six dimensions of SVH, ID, CD, SAFA, MC, and HI, respectively (12). A score below 60% of the total score (score < 40) is represented as low of HL (30). Therefore, in our study, we defined the cut-off points for adequate health literacy (HL) and low HL as being equal to or greater than 53 and less than 40, respectively.

### 2.5 Statistical analysis

First, participants were divided into two categories: (1) low HL (a total score < 40) and (2) basic HL (a total score ≥ 40). Second, categorical and continuous variables are expressed as numbers (percentages) and mean ± SD, respectively, testing normally distributed (Kolmogorov–Smirnov one-sample test, all *p*’s > 0.05). Second, we used chi-square tests for categorical variables to compare the differences between the low of HL and basic HL groups and ANOVA for continuous variables. Also, the LSD test was used to compare the differences between the means of multiple groups. Third, multiple linear regression was used to predict the factors influencing HL. The total score of the HLQ scale was used as the dependent variable, and demographic and socioeconomic variables were used as the independent variables. We used SPSS version 26.0 for all statistical analyses. All *p-*values were 2-tailed, with a significance level ≤ 0.05. Bonferroni corrections were used to adjust for multiple comparisons. Coefficient values, 95% confidence intervals, odds ratios, and were used to quantify the strength of correlation.

## 3 Results

### 3.1 Participants’ demographic characteristics

Of the 668 participants, 353 (52.8%) were male, and the mean age (SD) was 42.2 (12.7) years, with an age range of 15–77 years old. 657(98.4%) were the Wa, 92 (13.8%) were illiterate, and 294(44%) had an elementary school education level. The mean annual income was 47144.74 ± 40123.164 Chinese Yuan. The mean BMI was 24.13 ± 3.76 kg/m^2^, ranging from 15.90 to 39.96 kg/m^2^, of which 222 (33.2%) were overweight (BMI ≥ 24 kg/m^2^) and 102 (15.3%) were obese (BMI ≥ 28 kg/m^2^). The mean WC was 83.74 ± 10.72 cm, ranging from 52 to 114 cm, of which 247 (37%) were centrally obese.

### 3.2 Prevalence of adequate HL and low HL in participants

The mean score of HL was 29.19 ± 10.56, ranging from 2 to 60. According to the cutoff point of a HL total score ≥ 53 for adequate HL, 6 (0.89%) had adequate HL. In the three aspects of the HL scale, 45 (6.73%), 14 (2.1%), and 9 (1.34%) had adequate knowledge of KAA, BAL, and HRS, respectively. In the six dimensions of the HL scale, MC24 (3.59%), ID33(4.94%), CD36(5.39%), HI 44(6.59%), SVH 60(8.98%), and SAFA140(20.96%), and achieved adequate levels in MC, ID, CD, HI, SVH, and SAFA, respectively. Based on a total HL score < 40 as the cutoff point for low HL, 538 (80.68%) had low knowledge of HL. The participants were then divided into low HL and basci HL.

### 3.3 Comparison of the characteristics of participants between low HL and basic HL groups

As shown in Table 1, we observed that participants in the low health literacy (HL) group (*n* = 538), who had a total score < 40, were older and had higher body mass index (BMI) and waist circumference (WC) compared to the basic HL group (*n* = 129), who had a score ≥ 40. After Bonferroni correction (*p* < 0.05/16 = 0.003), age and WC remained significant (all *p*’s < 0.01). Also, there were significant differences between the two groups in the following variables: married status, sick leave, current smoking, normal weight, central obesity, education level, occupation, and residence. Meantime, pair comparisons were made for variables larger than three categories, including education levels, occupation, and residence. After Bonferroni correction (*p* < 0.05/9 = 0.006), education level, occupation, residence, and marital status remained significant (all *p*’s < 0.01). There were no significant differences between the groups in terms of gender, height, weight, and annual income (all *p* > 0.05). Further binary logistic regression showed that risk factors for low HL included education level (Wald = 20.852, *p* = 0.001), and residence (Wald = 30.811, *p* < 0.001) (see Table 2).

**Table 1:** The difference in socio-demographic and HL between Low HL and non-low HL groups.

Characteristic	Total participants (*N* = 668)	Low HL (*N =* 539) n(%)	Basic HL (*N* = 129) n(%)	χ^2^	*P*-value
Marital status					
Unmarried/Divorce	140(121)	98(18.2)_a_	42(32.6)_b_	27.355	0.000*
Married	528(79)	441(81.8)_a_	87(67.4)_b_		
Gender					
Male	353(52.8)	278(51.6)	75(58.1)	1.799	0.107
Female	315(47.2)	261(48.4)	54(41.9)		
Age (years)					
15~29	124(18.6)	83(15.4)	41(31.8)	35.73	<0.001***
30~44	247(37)	189(35.1)	58(45)		
45~59	241(36.1)	214(39.7)	27(20.9)		
60~69	51(7.6)	49(9.1)	2(1.6)		
70~69	5(0.7)	4(0.7)	1(0.8)		
70~79	5(0.7)	4(0.7)	1(0.8)		
Educational level					
Illiteracy	92(13.8)	87(16.1)_a_	5(3.9)_b_	68.866	<0.001***
Primary school	294(44)	260(48.2)_a_	34(26.4)_b_		
Junior school	200(29.9)	147(27.3)_a_	53(41.1)_b_		
Senior school	46(6.9)	29(5.4)_a_	17(13.2)_b_		
College or above	36(5.4)	16(3)_a_	20(15.5)_b_		
Residence				42.417	<0.001***
V1	96(14.4)	86(16.6)_a_	10(7.8)_b_		
V2	72(10.8)	60(11.1)_a_	12(9.3)_a_		
V3	84(12.6)	75(13.9)_a_	9(7)_b_		
V4	89(13.3)	84(15.6)_a_	5(3.9)_b_		
V5	84(12.6)	59(10.9)_a_	25(19.4)		
V6	75(11.2)	56(10.4)_a_	19(14.7)_a_		
V7	87(13)	67(12.4)_a_	20(15.5)_a_		
V8	81(12.1)	52(9.6)_a_	29(22.5)_b_		
Occupation					
Officer	11(1.6)	7(1.3)_a_	4(3.1)_a_	23.717	<0.001***
Healthcare	4(0.6)	2(0.4)_a_	2(1.6)_a_		
Student	27(4)	16(3)_a_	11(8.5)_b_		
Farmer	533(79.8)	449(84.2)_a_	84(65.1)_b_		
Worker	54(8.1)	38(7.1)_a_	16(12.4)_b_		
Others	39(5.8)	27(5)_a_	12(9.3)_a_		
Sick leave					
No	493(26.2)	387(71.8)	106(82.2)	7.360	0.025*
Yes	175(73.8)	152(28.2)	23(17.8)		
BMI				7.268	0.026*
Normal	344(51.5)	264(49)_a_	80(62)_b_		
Overweight	222(33.2)	187(34.7)_a_	35(27.1)_a_		
Obesity	102(15.3)	88(16.3)_a_	14(10.9)_a_		
Central obesity					
No	421(63)	328(60.9)	93(72.1)	5.643	0.018*
Yes	247(37)	211(39.1)	36(27.9)		
Smoking					
No	321(48.1)	248(46)	73(56.6)	4.666	0.031*
Yes	347(51.9)	291(54)	56(43.4)		

	Total participants (*N* = 668)	Low HL (*N* = 539) n(%)	Non-Low HL (*N* = 129) n(%)	*F*	*P*-value
Age (years)	42.192 ± 12.761	43.591 ± 12.715	36.349 ± 11.241	35.242	<0.001***
Educational level (years)	7.02 ± 3.807	6.469 ± 3.602	9.34 ± 3.780	64.896	<0.001***
Height	1.569 ± 0.079	1.567 ± 0.074	1.578 ± 0.097	2.118	0.146
Weight	59.397 ± 10.104	59.579 ± 9.909	58.636 ± 10.889	0.907	0.341
Income yearly (yuan)	47144.740 ± 40123.164	47601.600 ± 40745.953	45235.810 ± 37499.416	0.362	0.548
BMI (kg/m^2^)	24.129 ± 3.758	24.282 ± 3.766	23.490 ± 3.672	4.653	0.031*
WC (cm)	83.740 ± 10.722	84.360 ± 10.740	81.160 ± 10.289	9.383	0.002**
Total HL	29.907 ± 10.560	26.345 ± 8.257	44.791 ± 4.225	650.383	<0.001***
KAA	13.491 ± 5.267	11.896 ± 4.438	20.155 ± 2.511	414.597	<0.001***
BAL	10.130 ± 3.832	14.783 ± 2.362	9.017 ± 3.232	363.973	<0.001***
HRS	6.286 ± 2.998	5.432 ± 2.554	9.853 ± 1.880	342.025	<0.001***
SVH	4.868 ± 2.561	4.212 ± 2.235	7.612 ± 1.678	252.892	<0.001***
ID	2.781 ± 1.594	2.432 ± 1.441	4.279 ± 1.305	178.82	<0.001***
CD	5.395 ± 2.600	4.707 ± 2.276	8.271 ± 1.762	276.52	<0.001***
SAFA	7.832 ± 3.313	7.019 ± 2.816	11.233 ± 1.827	262.32	<0.001***
MC	6.177 ± 2.298	5.570 ± 1.963	8.713 ± 1.808	274.848	<0.001***
HI	2.847 ± 1.898	2.408 ± 1.758	4.682 ± 1.256	192.298	<0.001***

KAA, health knowledge; BAL, health-related behavior and lifestyle; HRS, health-related skills; SVH, basic and scientific health concepts; ID, infectious diseases prevention and control; CD, chronic disease prevention and control; SAFA, safety and first aid; MC, medical care; HI, health information. The significance of bold emphases in the table is *p* < 0.05. *P ≤ 0.05; **P ≤ 0.01; ***P ≤ 0.001. _a,b_Represent that the chi-square test was followed by a pairwise comparison of multiple groups, that is, the difference between two cells with the different subscripts is significant.

**Table 2:** Adjusted ORs of associated factors for low HL.

	B	S.E.	Wald	df	Sig.	Exp(B)	95% CI EXP(B)	
							Lower	Upper
Age	−0.016	0.011	1.871	1	0.171	0.984	0.963	1.007
Gender (male)	0.363	0.286	1.612	1	0.204	1.438	0.821	2.52
Smoking (yes)	0.389	0.283	1.892	1	0.169	1.475	0.848	2.566
Villages of residence			30.811	7	<0.001***			
V1	−1.767	0.465	14.414	1	0.001	0.171	0.069	0.425
V2	−1.1	0.47	5.485	1	0.019	0.333	0.133	0.836
V3	−1.567	0.475	10.899	1	0.001	0.209	0.082	0.529
V4	−2.332	0.55	17.959	1	0.001	0.097	0.033	0.285
V5	−0.525	0.395	1.769	1	0.183	0.592	0.273	1.282
V6	−0.728	0.396	3.377	1	0.066	0.483	0.222	1.05
V7	−0.742	0.366	4.118	1	0.042	0.476	0.233	0.975
Marital status			1.54	3	0.673			
Married	1.044	1.164	0.806	1	0.369	2.842	0.29	27.799
Unmarried	0.998	1.13	0.781	1	0.377	2.714	0.296	24.849
Divorce or others	0.204	1.401	0.021	1	0.884	1.226	0.079	19.091
Educational level			20.852	5	0.001***			
Illiterate	−2.86	0.879	10.593	1	0.001	0.057	0.01	0.321
Primary	−2.35	0.74	10.076	1	0.002	0.096	0.022	0.407
Junior high school	−1.57	0.725	4.69	1	0.03	0.208	0.05	0.862
Senior high school	−1.46	0.727	4.044	1	0.044	0.232	0.056	0.964
College and above	−0.21	0.76	0.076	1	0.783	0.811	0.183	3.599
Occupation			5.869	5	0.319			
Healthcare worker	−0.02	0.889	0	1	0.984	0.983	0.172	5.611
Student	0.597	1.311	0.207	1	0.649	1.816	0.139	23.696
Farmer	−0.88	0.683	1.643	1	0.2	0.417	0.109	1.589
Worker	−0.35	0.464	0.56	1	0.454	0.707	0.285	1.754
Others	0.356	0.545	0.428	1	0.513	1.428	0.491	4.152
BMI	−0.002	0.012	0.043	1	0.837	0.998	0.975	1.021
WC	−0.027	0.033	0.662	1	0.416	0.973	0.912	1.039
Constant	1.723	1.737	0.984	1	0.321	5.601		

BMI, body mass index; WC, waist circumference; OR, odds ratio. ***p < 0.001.

### 3.4 Relationship between HL and demographic characteristics

Table 3 summarizes the multiple linear regression models of HL predictors (*F* = 13.206, *p* < 0.0001, R2 = 0.290, adjusted R2 = 0.268). The model showed that HL significantly decreased with females (*β* = −2.225, *t* = −2.544, *p* = 0.0011), older age (*β* = −0.091, *t* = −2.644, *p* = 0.0008), current smoking (*β* = −2.66, *t* = −3.038 *p* = 0.019), and residence far from the local government office (*β* = −5.761, *t* = −4.1, *p* < 0.0001) and, however, HL significantly increased with residence closing to the local government office (*β* = 6.311, *t* = −4.096, *p* < 0.0001) and educational level (years) (*β* = 0.766, *t* = 0.262, *p* < 0.0001). However, no statistically significant differences were found in marital status, income yearly, BMI, WC, income yearly and occupation.

**Table 3:** Multivariate regression models for HL among Wa ethnic group.

	Unstandardized coefficients		Standardized coefficients	*t*	Sig.	95.0% confidence interval for B	
	B	SE	Beta			Lower bound	Upper bound
(Constant)	29.842	4.455		6.698	<0.0001	21.093	38.59
Gender (ref.male)	−2.225	0.874	−0.105	−2.544	0.011**	−3.941	−0.508
Age	−0.091	0.035	−0.11	−2.644	0.008**	−0.159	−0.023
Current smoking	−2.66	0.875	−0.126	−3.038	0.002**	−4.379	−0.941
WC	0.038	0.04	0.039	0.967	0.334	−0.04	0.116
Marital status (ref. unmarried)	−0.949	0.527	−0.066	−1.801	0.072	−1.985	0.086
educational level (years)	0.766	0.127	0.262	6.018	<0.001	0.516	1.016
Income yearly	1.46E-05	0	0.056	1.44	0.15	0	0
BMI	−0.024	0.108	−0.009	−0.221	0.825	−0.236	0.188
Village: V1	Ref.						
Village: V2	0.868	1.467	0.026	0.591	0.554	−2.014	3.749
Village: V3	−5.761	1.405	−0.181	−4.1	<0.001***	−8.519	−3.002
Village: V4	0.453	1.372	0.015	0.33	0.742	−2.242	3.147
Village: V5	4.194	1.442	0.132	2.909	0.004**	1.363	7.026
Village: V6	2.445	1.509	0.073	1.62	0.106	−0.518	5.409
Village: V7	3.263	1.496	0.104	2.181	0.03*	0.326	6.2
Village: V8	6.311	1.541	0.195	4.096	<0.001***	3.285	9.336
Occupation: farmer	Ref.						
Occupation: worker	1.746	1.377	0.045	1.268	0.205	−0.959	4.45
Occupation: offer/teacher	4.756	2.888	0.057	1.647	0.1	−0.914	10.426
Occupation: healthcare worker	4.989	4.668	0.036	1.069	0.286	−4.176	14.154
Occupation: student	2.451	2.089	0.046	1.173	0.241	−1.652	6.553
Occupation: other	0.447	1.58	0.01	0.283	0.777	−2.655	3.549

HL, health literacy; BMI, body mass index; WC, waist circumference. *P ≤ 0.05; **P ≤ 0.01,***P ≤ 0.001.

### 3.5 Comparison of HL among participants in different villages

**Figure 1:**
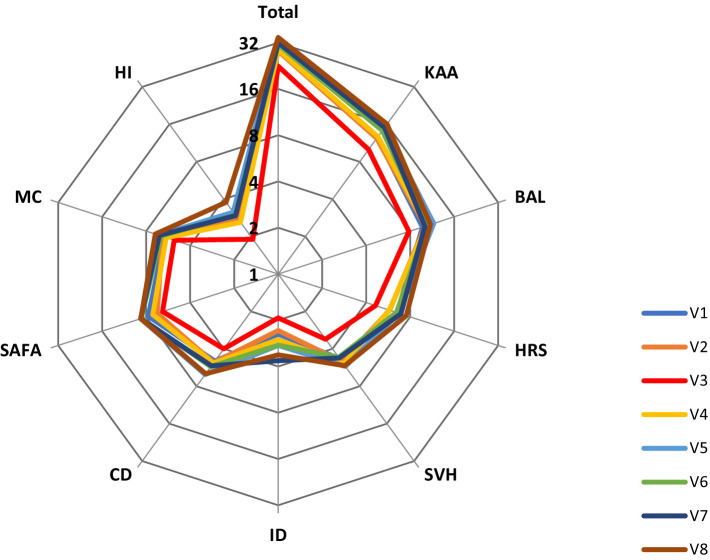
A summary of the health literacy total and nine domains’ scores of the eight villages.

We used ANOVA to analyze the differences in the HL total and all subscale scores between villages. From V1 to V8, there were significant differences in the total score of HL between eight villages (*F* = 11.578, *p* < 0.001). Further, using the LSD-test, we found that the HL in village V3 was significantly lower than the other seven villages (mean difference = −5.778–12.080, *p* < 0.001) and the HL in village V8 was significantly higher than in the other V1–V4 villages (mean difference = 5.749–12.080, *p* < 0.001). In addition, the distance from the village to the government office (from V1 to V8) were 2.7, 6.5, 15.2, 13, 7.5, 10, 13, and 0.2 km, respectively. There was a significant negative correlation between village HL scores and the distance between villages and government offices (*r* = −0.541, *p* < 0.001). Figure 1 shows the HL total score and nine domain scores for different villages (V1–V8).

## 4 Discussion

To our knowledge, this is the first study to investigate the prevalence of HL and associated factors in the Wa ethnic group. Our findings are as follows: (1) the prevalence of low HL among the Wa ethnic group was by far the highest among Chinese ethnic groups; and (2) demographic variables, including gender, age, education level, current smoking, and residence were associated with the low HL.

Up to date, there has been only one survey of HL among the Dulong, a “directly advancing” ethnic group in China, with a rate 1.46% adequate HL (18). Compared to this previous report, our result showed that the Wa group had the lowest rate of adequate HL (0.89%). Pu et al. reported that the adequate level of health knowledge, health-related behaviors and lifestyles, and health-related skills among the Dolong were 2.11, 3.14, and 0.16%, respectively, while the Wa ethnic group had adequate HL rates of 6.73%, 2.1 and 1.34%, respectively (18). These results suggest that there was a significant lack of health-related skills among these two “directly advancing” ethnic groups, which may be a key point for health promotion interventions.

Two main reasons may contribute to the low level of HL among the “directly advancing” ethnic groups. First, the Wa ethnic group and Dulong lived in mountainous areas until 1960 and maintained a slash-and-burn farming lifestyle. Due to natural, environmental, social, and historical issue (31, 32), ethnic groups tend to have low levels of HL and health status. Numerous studies have shown that high socioeconomic status (32), good education, and good living and working environments (33) weaken their exposure to risk factors affecting health and increase the likelihood of acquiring HL (34). Second, low HL is associated with limited knowledge of screening, lack of desire to screen, and language skills (35, 36). In this study, most villagers, especially the older adult, had low Chinese language proficiency, and their responses to the questionnaire required translation. However, there were no words or phrases in the Wa ethnic group language to name disease terms such as cancer, hypertension, hepatitis B, and tuberculosis. Some information was more likely to be lost in translation during the survey. Specifically, of the eight villages, Nanlang village was the most backward, being far from the government office and health center, with no road access to the village until 2016. Villagers using bilingual or the Wa ethnic group language at home with limited Chinese proficiency. Respondents in Nanlang village had significantly lower HL than those in other villages.

Our results are consistent with existing studies on ethnic groups. For example, in the United States, 44.9% of low language proficient ethnic groups reported limited English proficiency, while only 13.8% of fluent English speakers had low HL (37). More than half of Chinese Americans with limited English proficiency had low HL and cancer screening rates due to health communication barriers (37). These findings suggest that the inclusion of language proficiency in multivariate study factors associated with HL is critical to understanding all screening factors for low HL across ethnic groups, as limited language proficiency may compound vulnerabilities such as educational attainment, underutilization or misuse of health care resources, and decreased HL (38). Therefore, language proficiency associated with HL should be quantified to better understand the predictors of low HL, to inform future interventions, and to remove appropriate barriers to HL (39). There is growing evidence that multifaceted, culturally and linguistically appropriate interventions enable respondents with lower levels of health literacy to be more responsive (40, 41) such as the use of colloquial language in screenings and interviews.

To further explore the risk factors for low HL in the Wa ethnic group, our results showed that demographic information, including age, gender, education level, and village location was significantly associated with low HL through linear regression models. Previous studies have shown that the most common demographic characteristics related to HL were education, age (31), gender (42), ethnicity (43), residence (44, 45), and income (45, 46). The meta-analysis study reported that of these variables, age, education, and race were the most consistently included in the regression equation (47). Nearly all of our respondents had a low level of education, with 87.7% having an elementary or middle school level. Illiteracy accounted for 13.8% of the population, and the mean age of illiterates was 52 ± 10.44 years, of which 62% were female. This is due to limited educational opportunities for Chinese seniors at a young age, resulting in low education levels and even illiteracy (48). Women typically have less access to higher education than men (49). An individual’s low educational background often leads to negative attitudes toward health management (50–53), affecting HL and impacting access to health care services.

Besides, several barriers, such as geography, distance (52), bad weather (29), and rural or urban (15), contribute to low levels of HL. Rural–urban differences in health information sources may be due to structural barriers, such as a shortage of rural specialists, inadequate online resources (51), and greater distance from the nearest health facility (53). Patients in rural areas are two to three times further away from medical appointments than those in urban areas, resulting in residents potentially having fewer opportunities to ask for or receive health information from specialists (54). Similarly, among our participants, those who were farther away from the township office (mean distance of 32 m) had lower HL than those closer to the township office (mean distance of 18 m). In each village studied, the township government office, health center, and other facilities are all centralized in the same or adjacent buildings. The township government office serves as a political, cultural, and informational center, providing many conveniences for villagers and offering health or political information resources. These results also illustrated that improving access to health information or other resources within the community is key to improving HL.

Few studies highlighted the relationship between BMI or WC and HL. Some studies have found a strong correlation between low HL and obesity (55), whereas others have shown that HL did not predict BMI (56). Similarly, our study showed that the association between BMI or WC and HL was statistically significant but not a risk predictor. 37 and 48.1% of our respondents who were centrally obese and smokers, respectively, had significantly lower HL than nonsmokers and nonobese individuals. Higher HL levels were associated with higher access to health information and lower risky habits, such as physical inactivity, smoking, and alcohol consumption (57, 58). These results may be explained by the strong relationship between a person’s BMI or WC and lifestyle, as those with low HL are more likely to have unhealthy diets and behaviors (59). Evidence suggests that effective interventions to improve HL in community settings may be to change unhealthy lifestyles (e.g., smoking, diet, and physical activity) (60). Therefore, we recommend that rural health professionals be trained to properly perform WC measurements and consider them an important and more accurate “vital sign” in screening goals to improve HL (61).

### 4.1 Strengths and limitations

This is the first study to examine the HL among the Wa ethnic group and its influencing factors. However, there are also several limitations. First, our study data came from the Wa ethnic group along the China-Burma border. Some compounding factors, such as economic, cultural, geographic environment, and health status conditions, were not taken into account; therefore, our results should be examined and carefully generalized to other studies from different cultural backgrounds. Second, the limited Chinese language proficiency of our participants may have influenced their responses to the HL questionnaire. Therefore, further research on HL among ethnic groups should consider using different data collection methods than survey studies. Finally, the data were measured by using a screening scale from a survey and thus it is difficult to avoid recall bias.

## 5 Conclusion and relevance for practice

In summary, this study is the first to examine HL among the Wa, a “directly advancing” ethnic group in China. Significant associations exist between low HL and age, gender, education level, place of residence, and current smoking. In particular, lower education level and residence far from the township were risk factors for low HL. These factors, including senior, female, smoking, low education, and living far from the township, have been identified as risk factors for HL. This finding helps to clarify the goals for improving HL and to develop problem-oriented health education among the Wa population.

## Data availability statement

The original contributions presented in the study are included in the article/supplementary material, further inquiries can be directed to the corresponding authors.

## Ethics statement

The studies involving humans were approved by the Review Board (IRB) of the Institute of Psychology, Chinese Academy of Sciences (Beijing, China). The studies were conducted in accordance with the local legislation and institutional requirements. The participants provided their written informed consent to participate in this study.

## Author contributions

WY: Conceptualization, Methodology, Supervision, Writing – original draft, Writing – review & editing. YL: Conceptualization, Data curation, Investigation, Methodology, Writing – original draft. GZ: Conceptualization, Investigation, Project administration, Resources, Writing – original draft. YY: Formal analysis, Investigation, Project administration, Writing – original draft. YW: Investigation, Project administration, Writing – original draft. DL: Investigation, Project administration, Software, Writing – original draft. CL: Data curation, Investigation, Software, Writing – review & editing. KL: Data curation, Investigation, Software, Writing – original draft. JL: Investigation, Software, Writing – review & editing. YP: Investigation, Writing – review & editing. ML: Investigation, Writing – review & editing. BY: Investigation, Writing – review & editing. SZ: Investigation, Writing – review & editing. DM: Investigation, Writing – review & editing. XZ: Supervision, Writing – review & editing.
